# IL-12p40/IL-10 Producing preCD8α/Clec9A^+^ Dendritic Cells Are Induced in Neonates upon *Listeria monocytogenes* Infection

**DOI:** 10.1371/journal.ppat.1005561

**Published:** 2016-04-13

**Authors:** David Torres, Arnaud Köhler, Sandrine Delbauve, Irina Caminschi, Mireille H. Lahoud, Ken Shortman, Véronique Flamand

**Affiliations:** 1 Institut d’Immunologie Médicale, Université Libre de Bruxelles, Gosselies, Belgium; 2 Centre for Biomedical Research, Burnet Institute, Melbourne, Victoria, Australia; 3 Department of Microbiology and Immunology, The University of Melbourne, Parkville, Australia; 4 Department of Immunology, Monash University, Melbourne, Australia; 5 The Walter and Eliza Hall Institute of Medical Research, Parkville, Australia; 6 Department of Medical Biology, University of Melbourne, Parkville, Australia; Duke University School of Medicine, UNITED STATES

## Abstract

Infection by *Listeria monocytogenes* (*Lm*) causes serious sepsis and meningitis leading to mortality in neonates. This work explored the ability of CD11c^high^ lineage DCs to induce CD8^+^ T-cell immune protection against *Lm* in mice before 7 days of life, a period symbolized by the absence of murine IL-12p70-producing CD11c^high^CD8α^+^ dendritic cells (DCs). We characterized a dominant functional Batf3-dependent precursor of CD11c^high^ DCs that is Clec9A^+^CD205^+^CD24^+^ but CD8α^-^ at 3 days of life. After *Lm*-OVA infection, these pre-DCs that cross-present Ag display the unique ability to produce high levels of IL-12p40 (not IL-12p70 nor IL-23), which enhances OVA-specific CD8^+^ T cell response, and regulatory IL-10 that limits OVA-specific CD8^+^ T cell response. Targeting these neonatal pre-DCs for the first time with a single treatment of anti-Clec9A-OVA antibody in combination with a DC activating agent such as poly(I:C) increased the protection against later exposure to the *Lm-*OVA strain. Poly(I:C) was shown to induce IL-12p40 production, but not IL-10 by neonatal pre-DCs. In conclusion, we identified a new biologically active precursor of Clec9A^+^ CD8α^-^ DCs, endowed with regulatory properties in early life that represents a valuable target to augment memory responses to vaccines.

## Author Summary

*Lm* is a gram-positive food-borne pathogen that is the ethiological agent of listeriosis, a worldwide disease reported most frequently in developed countries. It can cause spontaneous septic abortions, fatal meningitis or encephalitis in immunocompromised and pregnant individuals. The murine model of systemic *Lm* infection has been demonstrated as a useful model to understand host resistance to intracellular pathogens. Neonates are highly susceptible to infections such as *Lm*, and display low responses to vaccines requiring IFN-γ producing T cells. In the present study, we characterized in murine neonates a precursor of conventional dendritic cells that is able to produce IL-12p40 and IL-10 cytokines and to modulate the development of the adaptive immune response, more particularly the CD8^+^ T cell response upon exposure to *Lm*. By targeting *Lm*-associated antigens to these conventional dendritic cell precursors in neonates, we succeeded to confer a partial protection to a lethal dose of *Lm* at the adult stage. Our study provides new insights into our understanding of the innate immune response to infections in early life and will help to design new vaccine strategies in newborns.

## Introduction

Early life is a period of immune maturation characterized by a high susceptibility to infectious diseases. The underdeveloped immune system gives a Th2-biased response and has an impaired ability to develop long-lasting protective CD8^+^ T cell immunity [1, 2]. We are particularly interested in immune resistance to infections by *Listeria monocytogenes (Lm)*. *Lm* is a gram-positive opportunistic food-borne bacteria with a facultative intracellular life cycle that commonly causes sepsis and/or meningitis, leading to mortality in neonates but is asymptomatic in immunocompetent *Lm*-infected adults [3].

DCs are the key components of the immune system, determining susceptibility to infections. The primary function of DCs is the detection of pathogens and the initiation of the adaptive immune response. Such a response requires the DCs to present an antigen (Ag) from a specific pathogen, as well as an innate signal from microbes or damaged cells allowing DCs to orchestrate the adaptive immune response. Conventional DCs (cDCs) in mice can be divided into two distinct populations, one with high expression of CD8α (CD8α^+^ cDCs) and the other with no expression of CD8α (CD8α^-^ cDCs). These CD8α^+^ cDCs selectively express the C-type lectin receptor DNGR1, also called Clec9A [4]. The development of CD8α ^+^ cDCs depends on a common set of transcription factors including Irf8 [5], Batf3 [6], Id2 [7] and Nfil3 [8]. CD8α^+^ cDCs are particularly efficient at internalizing and cross-presenting exogenous Ag on MHC class I molecules, especially from dead or dying cells [9–14]. cDCs represent a key subset which initiates cell-mediated immunity against tumors, viruses and bacteria [15, 16]. Upon *Lm* infection, adult CD8α ^+^ DCs phagocytize the bacteria in the marginal zone of the spleen, and migrate to the T-cell zone in order to present the bacterial antigens to CD8^+^ T cells [17]. The resultant response involves the up-regulation of co-stimulatory molecules, the production of cytokines like IFN-γ and the generation of cytotoxic T-cell immunity. Finally, CD8α^+^ cDCs have been identified as professional IL-12p70 producers priming the adaptive immune cells towards Th1 differentiation [18–21].

In murine neonates, CD8α^+^ cDCs have been shown to be defective in the first 6 days of life. Beyond this time, the CD8α^+^ cDCs producing IL-12p70 induces the downregulation of the IL-4Rα/IL-13Rα1 on T cells, favoring a Th1 response [2]. Since the study by Lee H. et al. [2], the immune neonatal period has been redefined. As a result, some of the previous reports on the quantitative and qualitative shortcoming of neonatal DCs have to be revisited. For example, it was demonstrated that at 7 days of life the Flt3 ligand-treated “neonatal” mice showed an increase in DCs lineage development and an increased in IL-12-dependent innate resistance against *Lm* [22]. Another study reported that one-day-old DCs were able to produce adult level of IL-12p70, but only after IL-4, a maturating cytokine, was added to GM-CSF and CpG in the culture [23].

Neonatal induction of Th1/Tc1 memory is still controversial. Neonates have shown to be more susceptible to intracellular pathogens due to a suboptimal capacity to mount an efficient cell-mediated immunity, particularly the memory CD8^+^ T cells. For instance, qualitative defect in neonatal Batf3-dependent CD103^+^ lung DCs were recently reported to influence the CD8^+^ T cell response, following respiratory syncytial virus (RSV) infection [24]. However, other studies have demonstrated that neonates could mount an adult-like CD8^+^ T cell immune response against human CMV or *Trypanosoma cruzi* [25, 26]. Concerning *Lm* infection in early life, a previous study demonstrated that 5- to 7-days old neonates are able to develop robust primary and secondary CD4^+^ and CD8^+^ Th1-type responses against *L*m without characterizing the antigen presenting cells that were involved [27].

The objectives of this study were to describe the phenotype of Batf3-dependent CD11c^high^ DC subset and to explore their abilities to induce a CD8^+^ T cell immune protection against *Lm* at 3 days of life. First, we characterized the splenic DC subset bearing DNGR1/Clec9A but not CD8α, a precursor of CD8α ^+^ DCs. This DNGR1/Clec9A bearing DC is the predominant lineage before 6 days of life. Next, we demonstrated the role of these early DCs in taking up and presenting exogenous *Lm* Ag to prime a CD8^+^ T-cell response. Additionally, we defined the role of IL-12p40 and IL-10 uniquely produced by these neonatal pre-DCs in the establishment of an adaptive response. Finally, we assessed vaccination strategies, directly targeting neonatal DCs using OVA coupled to anti-Clec9A in the presence of poly(I:C). This study clarifies the function of pre-CD8α ^+^ DCs in early life and highlights the advantages for human neonatal vaccination strategies.

## Results

### Neonatal Batf3-dependent DCs are required to induce T-cell response against *Lm*

**Fig 1:**
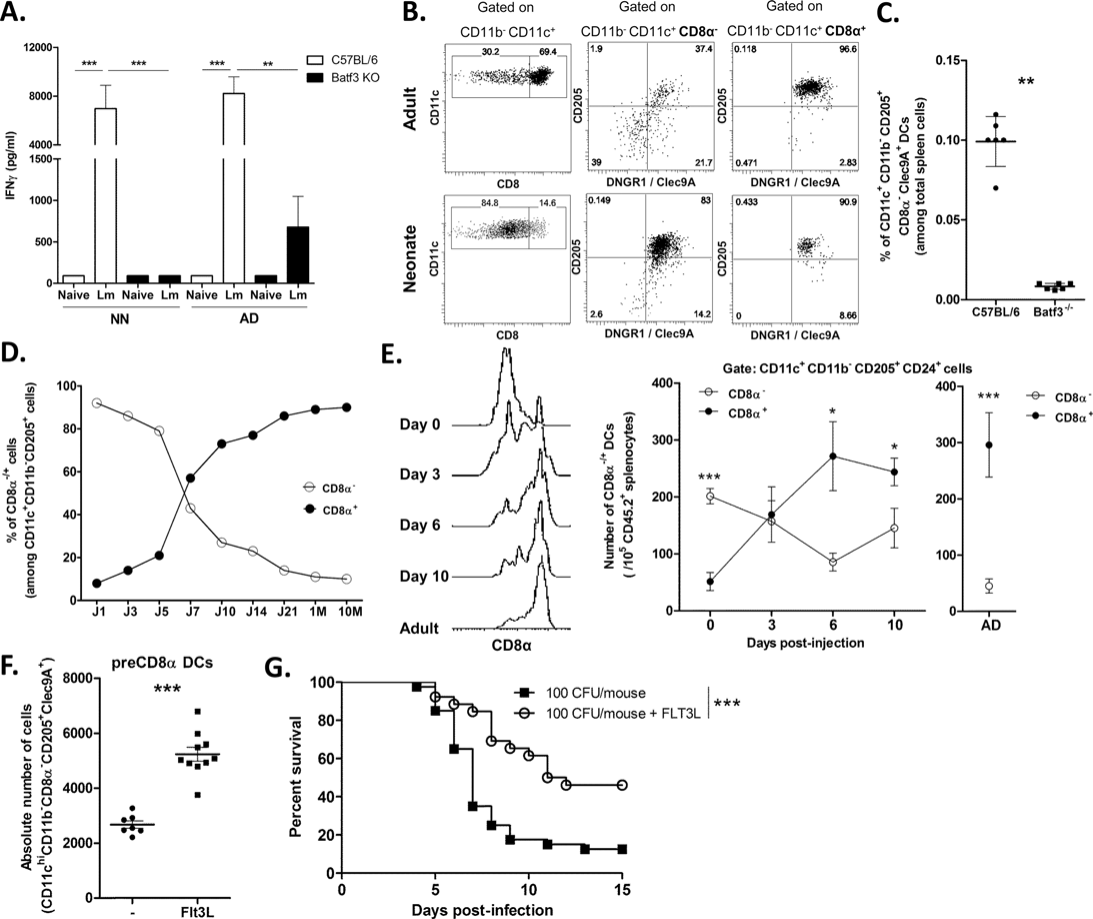
Identification of splenic precursors of CD8α^+^ DCs in neonates. A, 3-day-old (NN) or adult (AD) spleen cells (10^6^) harvested from C57BL/6 (white) or Batf3^-/-^ (black) mice 7 days after Lm actA^-/-^-OVA *i*.*p*. injection (5.10^5^ CFU) were incubated with OVA^257-264^ peptide. Production of IFN-γ was measured by ELISA in the supernatant and normalized to 10^5^ CD8^+^ T cells (n = 8/group). Results are a pool of 3 independent experiments. B, Spleen cells of C57BL/6 adults and neonates were harvested, stained with anti-CD11c, -CD11b, -CD8α, -CD205 and -Clec9A mAbs and analyzed by flow cytometry. Numbers indicate percentage of cells in the gate. Representative of 6 independent experiments. C, Frequencies of splenic CD11c^+^CD11b^-^CD8α^-^CD205^+^Clec9A^+^ DCs in C57BL/6 and Batf3^-/-^ neonates. Dot Plot and graphs for the percentages of CD11c^+^CD11b^-^CD8α^-^CD205^+^Clec9A^+^ cells of individual mice are shown. D, Spleen cells of C57BL/6 mice from birth to adulthood were collected and analyzed by flow cytometry for the percentage of CD8α^-^ and CD8α^+^ cells within the CD11c^+^CD11b^-^CD205^+^ DCs fraction. E, Adoptive transfer. Spleen cells of C57BL/6 CD45.2^+^ neonates were collected and depleted in CD3^+^, CD19^+^ and Gr1^+^ cells by negative selection. Enriched cells were *i*.*v*. injected into C57BL/6 CD45.1^+^ adult mice. Representative expression (single histograms) of CD8α and numbers of CD8α^+^ and CD8α^-^ cells among 10^5^ spleen cells were analyzed by flow cytometry at day 0, 3, 6 and 10 after transfer (n = 3-4/timing), staining on adult CD45.2 spleen cells are shown as comparison. F. Absolute numbers of splenic CD11c^+^CD11b^-^CD8α^-^CD205^+^Clec9A^+^ DCs in saline buffer- (n = 7) or Flt3L-treated C57BL/6 3-day-old mice (n = 10). G. C57BL/6 neonates (n = 18) were injected s.c. with Flt3L (20μg/mL) or with saline buffer. At day 3, neonates were infected with 100 CFU of *Lm* and monitored daily for survival.

To determine the type of DC involved in the adaptive immune response against *Lm* at 3 days of life, we employed Batf3^-/-^ mice known to lack the conventional CD8α^+^ type of DC [6]. We compared the OVA-specific primary immune response to the attenuated strain *Lm* actA^-/-^-OVA in 3-day-old and adult C57BL/6 and Batf3^-/-^ mice. As seen in Fig 1A, the production of IFN-γ, following restimulation with MHC I-restricted OVA peptides, was drastically reduced in splenic cultures from Batf3^-/-^ mice compared with C57BL/6 mice at both ages. This suggested that Batf3-dependent DCs are required to trigger an IFN-γ T cell response against *Lm* infection at 3 days of life as well as at the adult stage.

### Characterization of Batf3-dependent DCs in the neonatal spleen

To characterize the DCs likely to generate an adaptive CD8^+^ T-cell response against *Lm* in neonates, we focused on the Batf3-dependent CD8α^+^ DC lineage. In naïve adult C57BL/6 spleen, the majority (69%) of CD11b^-^CD11c^high^ cells were CD8α^+^ (Fig 1B). In contrast, in neonates, the majority (85%) of splenic CD11b^-^CD11c^high^ cells lacked CD8α expression but were positive for CD205 and DNGR1/Clec9A, which are features of CD8α^+^ DC family [4], as confirmed in adult splenic CD8α^+^ DCs (Fig 1B). We further observed that CD11c^high^ CD11b^-^CD205^+^DNGR1^+^ DCs in mesenteric lymph nodes collected from 5 day-old C57BL/6 mice (S1 Fig) expressed the same intermediate level of CD8α as their splenic counterpart. The neonatal splenic CD11c^high^ CD11b^low^ CD205^+^CD8α^-^ DCs also expressed CD24 and cKIT at similar levels but expressed MHCII, CD80 and CD86 molecules at a lower level, compared to adult CD8α ^+^ DCs (S2A Fig). Interestingly, neonatal CD11c^high^CD11b^low^CD205^+^CD8α ^+^ or CD8α ^-^ displayed similar levels of aforementioned molecules suggesting that they were closely related phenotypically (S2A Fig). No expression of CD207, CD4 or B220 was detected in neonatal CD8α^-^CD11b^-^CD205^+^ DCs nor in adult CD8α^+^CD11b^-^CD205^+^DCs (S2A Fig) and the high expression of DNGR1/Clec9A was restricted to neonatal CD8α ^-^CD11b^-^CD205^+^ DCs and not to neonatal pDCs or cDC2 (S2B Fig). These results demonstrated that neonatal CD8α ^-^CD11b^-^ DCs display a surface phenotype closely similar to adult CD8α ^+^ DCs except for the expression of CD8α, what suggests that they are earlier forms of the same DC lineage. This was supported by analysis of Batf3^-/-^ neonates in which no CD11c^+^CD11b^-^CD205^+^DNGR1^+^ CD8α ^-^ DCs could be detected (Fig 1C and S3A Fig).

Next we examined the developmental relationship between neonatal CD11b^-^CD8α ^-^ DCs and adult CD8α ^+^ DCs. A time course of the frequency of CD11c^+^CD11b^-^CD205^+^CD8α ^-^ DCs versus CD11c^+^CD11b^-^CD205^+^CD8α ^+^ DCs showed that the latter were dominant for the first 5 days of life. After day 7, the proportions were totally reversed as CD11c^+^CD11b^-^CD205^+^ DCs expressing CD8α became dominant (Fig 1D). We thus confirmed the appearance and accumulation of the CD8α ^+^ DCs at day 6 as previously reported [2]. Finally, to test if neonatal spleen contained precursors of the CD8α ^+^ DC type found in adults, we transferred, into adult C57BL/6 CD45.1^+^ mice, neonatal C57BL/6 CD45.2^+^ spleen cells that were depleted of CD3^+^, CD19^+^ and Gr1^+^ cells and that contained mostly CD11c^+^CD11b^-^CD24^+^ CD205^+^ CD8α ^-^ cells. The number of DCs of donor origin expressing CD8α among CD45.2^+^CD11c^+^ CD11b^-^ CD24^+^ CD205^+^ spleen cells showed a steady increase to adult levels by day-6 post transfer (Fig 1E). Taken together, these results suggested that earlier forms of the CD8α ^+^ DCs, that we will call preCD8α Clec9A^+^ DCs, are predominant among splenic and mesenteric lymph nodes CD11c^high^ DCs from birth to 5 days of life.

We further determined that C57BL/6 neonates that were submitted to a 3 day treatment with the dendritic cells growth factor, Flt3L [28] (instead of 7 days as previously reported [22]), expanded preferentially the absolute number of preCD8α Clec9A^+^ DCs (Fig 1F) without affecting the number of neonatal plasmacytoid DCs or conventional DC2 (S3C Fig) and significantly enhanced their defense against *Lm* (Fig 1G) as previously described [22].

### Functional capacity of neonatal preCD8α Clec9A^+^ DCs

**Fig 2:**
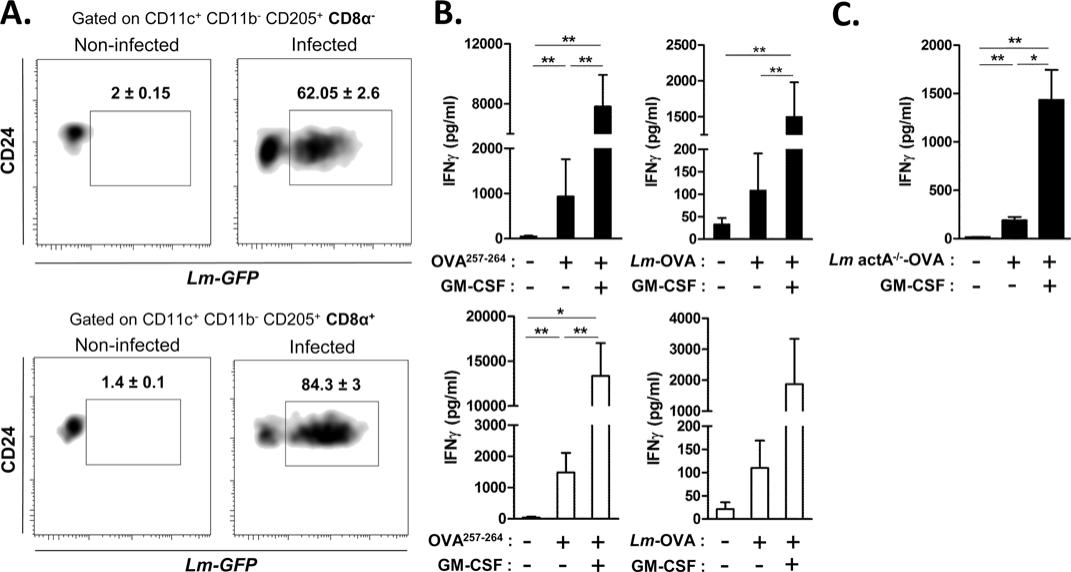
Function of neonatal preCD8α Clec9A^+^ DCs. A, Neonatal or adult spleen cells were cultured with *Lm*-GFP (MOI 1:5). After 2h, spleen cells were stained, gated on CD11c^+^CD11b^+^CD205^+^CD24^+^CD8α^-^ for neonates or CD8α^+^ for adults and phagocytosis of *Lm*-GFP was analyzed by FACS. Dot plot is representative of 6 independent experiments. Percentages are shown with SD. B, Neonatal CD11c^+^CD11b^-^CD205^+^CD24^+^CD8α^-^ DCs (black histograms) or equivalent adult CD8α^+^ DCs (white histograms) were sorted and stimulated with OVA257-264 peptide (SIINFEKL; 1μg/mL) or Lm-OVA (MOI 1:5) with or without GM-CSF (20ng/mL). OT-I T cells was added to the culture (ratio 1 DC to 5 OT-I) and IFN-γ was measured by ELISA (n = 4-5/group). C, C57BL/6 neonates were i.p. injected with Lm actA^-/-^-OVA (10^6^ CFU). After 24h, CD8α^-^ DCs were sorted and co-cultured with OT-I T cells (ratio 1 DC to 5 OT-I), with or without GM-CSF (20ng/mL). IFN-γ was measured by ELISA (n = 4-6/group).

As presented in Fig 2A, we first demonstrated that neonatal preCD8α Clec9A^+^ DCs were able to phagocytize *Lm*-GFP (62%±2.6) as did adult CD8α ^+^ DCs (84.3%±3). To assess capacity to cross-present cell-associated Ag during *Lm* infection, we purified splenic C57BL/6 neonatal preCD8α Clec9A^+^ DCs or adult CD8α ^+^ DCs, incubated them with OVA_257-264_ peptides or *Lm*-OVA and co-cultured them with OVA-specific OT-I T cells, with or without GM-CSF known to enhance cross-presentation capacity of newly formed DC [29]; and monitored the IFN-γ production (Fig 2B). Neonatal preCD8α Clec9A^+^ DCs were able to cross-present OVA as efficiently as adult CD8α ^+^ DCs in the presence of GM-CSF. We evaluated at the RNA level the cDNA relative expression of a large number of genes involved in the cross-presentation machinery (such as Rac2, Ergic 1, 2 and 3, Rab14, Erap1, Sec22b, Syntaxin 4, TAP1 and 2) and concluded that they were similar in sorted preCD8α DCs and adult CD8α DCs except for a slight difference with H2-K1, β2m and Rab27a (S4 Fig). This cross-presentation ability was confirmed *ex vivo* with neonates that were injected with *Lm* actA^-/-^-OVA, from which preCD8α Clec9A^+^ DCs were sorted and co-cultured with OT-I T cells, with or without GM-CSF (Fig 2C).

**Fig 3:**
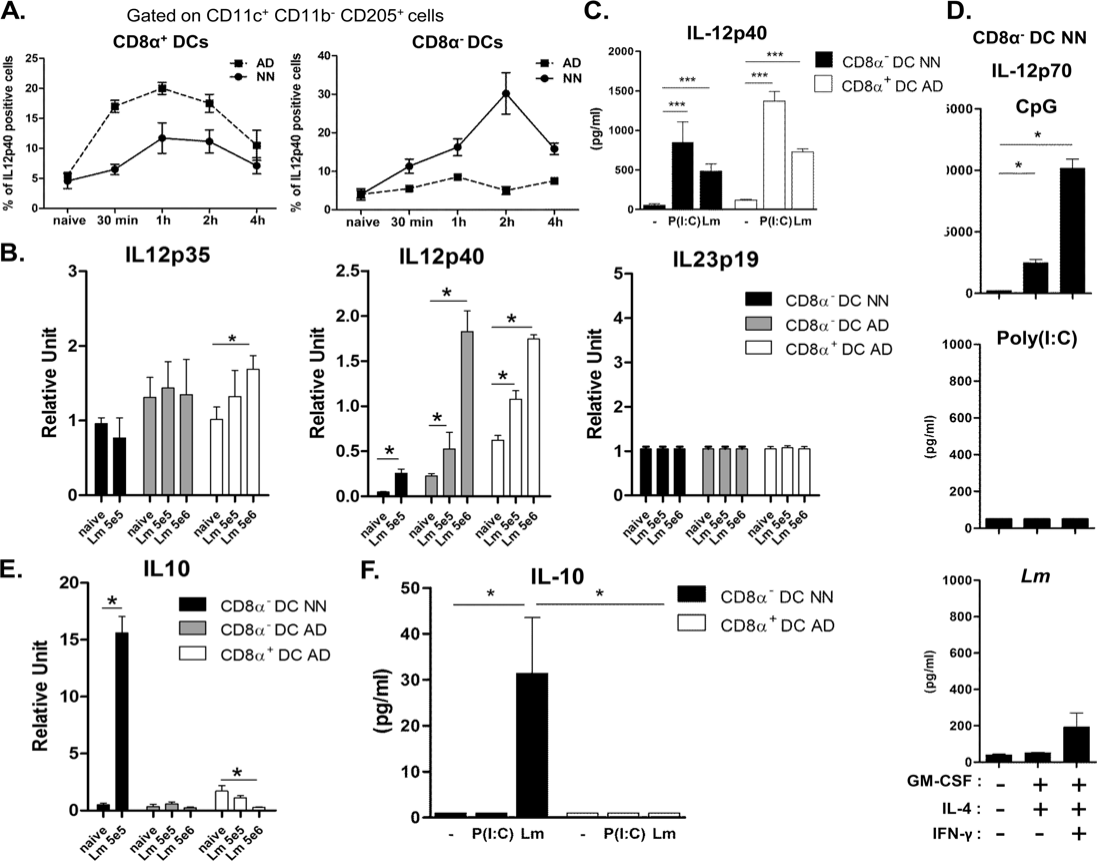
Neonatal preCD8α Clec9A^+^ DCs produce IL12p40 and IL-10. A, C57BL/6 neonates and adults were *i*.*v*. injected with poly(I:C) (1 mg/kg). Percentages of IL-12p40-producing CD8α^-^ and CD8α^+^ DCs (gated on CD11c^+^CD11b^-^CD205^+^ cells) were evaluated by flow cytometry (n = 3-4/timing). B and E, mRNA normalized expression of IL-12 family genes (IL-12p35, IL-12p40 and IL-23p19 (B) and IL-10 (E) from CD8α^-^ DCs or CD8α^+^ DCs sorted from spleen of neonates and/or adults injected or not with 5 x 10^5^ or 5 x 10^6^ CFU *Lm* actA^-/-^ for 24h were analyzed by quantitative real-time PCR. Relative unit was obtained by comparing each group to 5 x 10^5^ CFU *Lm* actA^-/-^ -infected adults (n = 4-8/group). C, D and F, sorted neonatal CD8α^-^ and/or adult CD8α^+^ DCs were simulated *in vitro* with poly(I:C) (10 μg/mL), *Lm* (MOI 1:1) or CpG (2 μg/ml). IL-4, GM-CSF and IFN-γ were added when indicated. IL12p40 (C), IL12p70 (D) and IL-10 (F) were measured by ELISA (n = 4-6/group).

We then assessed the capacity of neonatal preCD8α Clec9A^+^ to produce cytokines in several settings such as *Lm* exposure or TLR ligand encounter and compared it to fully competent adult CD8α ^+^ DCs. As an endosomial TLR receptor, TLR3 binds to ligand such as dsRNA, namely polyinosinic-polycytidylic acid (Poly(I:C)) which mimic the replication intermediate of virus. TLR3 agonist was shown to promote the cross-presentation of antigens that transit in the endosome as it increases MHC class I and costimulatory molecules of DCs and stimulate IL-12 secretion. It is considered as an efficient manner to optimally activate DCs to promote CD8^+^ T cell activation. Moreover, TLR3 is only expressed in CD8α ^+^ DC subset. Poly(I:C) was first injected into C57BL/6 adults and neonates and intracellular staining for IL-12p40 was performed on CD11c^+^CD11b^-^CD205^+^CD8α ^+^ and CD11c^+^CD11b^-^CD205^+^CD8α ^-^ at different time points (Fig 3A and S5 Fig). As expected, the overall number and relative frequency of IL-12p40 producing CD8α ^+^ DCs was higher in adults than in neonates. Interestingly, 2h after poly(I:C) treatment, a high proportion of CD8α ^-^ DCs were producing IL-12p40 in neonates but not in adults (Fig 3A).

To assess cytokine responses by the DC to *Lm* infection, cytokine secretion of cultured cells and mRNA synthesis was utilized. For mRNA measurement, we sorted DC subsets from neonates infected with 5 x 10^5^ *Lm* actA^-/-^ or adults infected with 5 x 10^5^ or 5 x 10^6^ CFU *Lm* actA^-/-^. IL-12p35 mRNA synthesis was induced in adult CD8α ^+^ DCs but not in CD8α ^-^ DCs at both ages (Fig 3B). A significant induction of IL-12p40 mRNA synthesis was observed in neonatal and adult CD8α^-^ DCs and in adult CD8α ^+^ DCs upon *Lm* actA^-/-^ infection (Fig 3B). In addition, sorted neonatal preCD8α Clec9A^+^ DCs secreted quite similar protein levels of IL-12p40 as adult CD8α ^+^ DCs after *in vitro* stimulation with poly(I:C) or *Lm* (Fig 3C). However, neither IL-12p70 (Fig 3D) nor IL-23 (S6 Fig) were significantly produced by these neonatal preCD8α Clec9A^+^ DCs after such stimulation, even in the presence of GM-CSF, IL-4 and IFN-γ; by contrast, CpG did induce IL-12p70 secretion in such maturating conditions (Fig 3D) as previously described [23]. IL-23p19 mRNA synthesis was not induced in all the tested DC subsets in response to *Lm* infection (Fig 3B). Of particular interest, IL-10 transcripts were strongly induced only in neonatal preCD8α Clec9A^+^ DCs after *Lm* actA^-/-^ infection whereas adult preCD8α Clec9A^+^ DC and adult CD8α ^+^ DCs did not (Fig 3E). The exclusive production of IL-10 by neonatal preCD8α Clec9A^+^ DCs after *Lm* actA^-/-^stimulation was confirmed *in vitro* at the protein level (Fig 3F). Finally, the TLR expression was compared between preCD8α DCs and CD8α ^+^ DCs to potentially explain their distinct functionality. As presented in S7 Fig, TLR9 RNA expressions were similar and TLR3 RNA was expressed only 1.14 times more in adult CD8α ^+^ DCs. Altogether, these results indicate that, upon *Lm* infection, splenic neonatal preCD8α Clec9A^+^ DCs have the unique ability to produce IL-12p40 and IL-10 but no IL-12p70 and IL-23.

### IL-12p40 and IL-10 secreted by neonatal preCD8α Clec9A^+^ DCs influenced the CD8^+^ T-cell response against *Lm*

**Fig 4:**
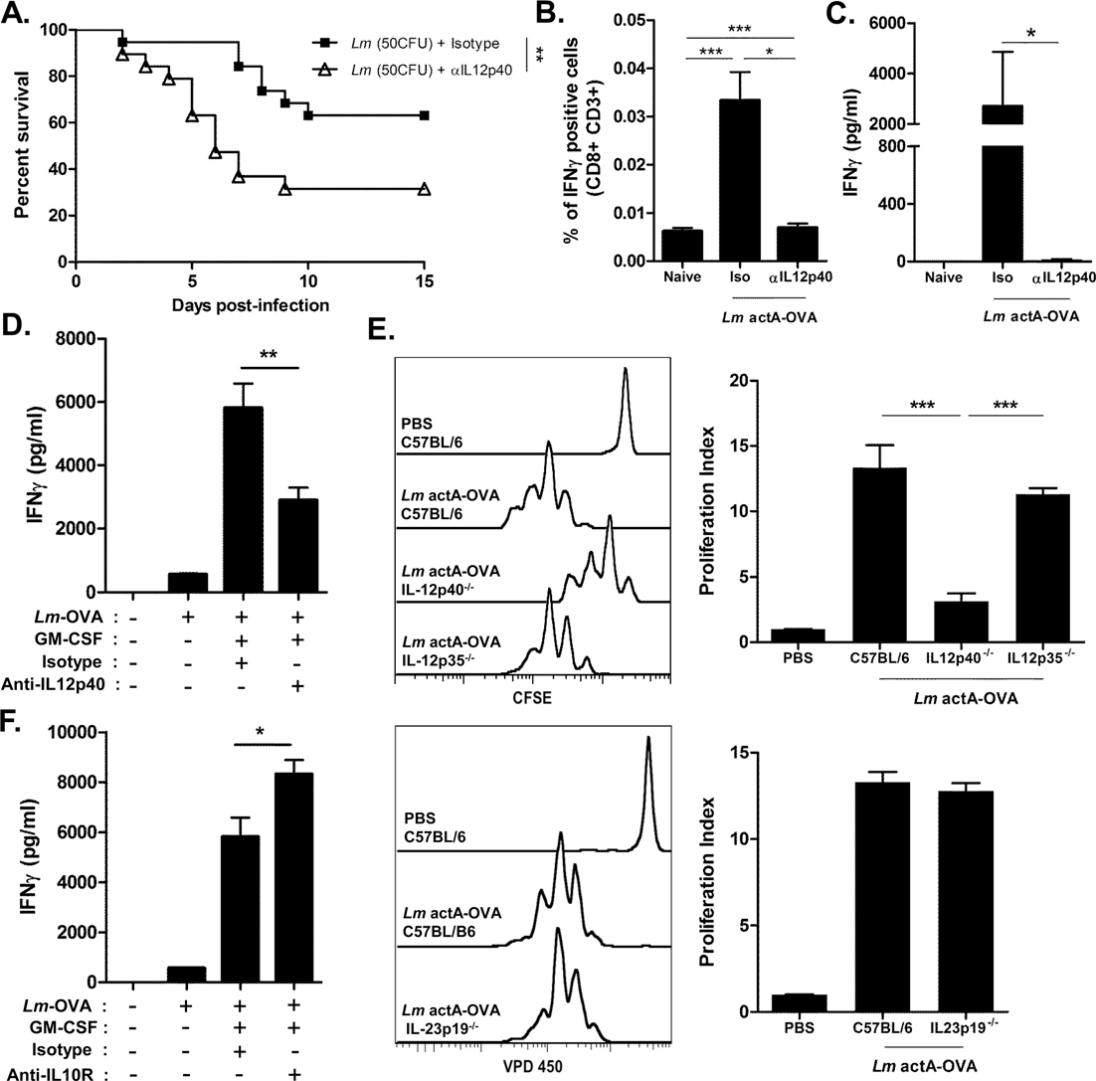
Role of IL-12p40 and IL-10 in immune response against *Listeria*. A, C57BL/6 neonates injected with anti-IL12p40 mAb or isotype mAb (25 μg/neonate; day 0, 1 and 3) were infected with *Lm* (50 CFU) and followed for survival (n = 12/group). (B-C) Neonates were injected with *Lm* actA^-/-^-OVA (5.10^5^ CFU) with or without anti-IL12p40 mAb. Spleen cells were collected 7 days later and cultured with OVA^257-264^ peptide. B. CD8^+^CD3^+^ cells were analyzed for IFN-γ production by flow cytometry (n = 5/group). C, IFN-γ was measured in the culture supernatants by ELISA (n = 5/group). D and F, C57BL/6 neonatal CD8α^-^ DCs were sorted and cultured with *Lm*-OVA (MOI 1:1) for 4h and OT-I T cells (at a ratio 1 DC to 5 T cells). GM-CSF (20ng/mL), anti-IL12p40 (D) or anti-IL-10R (F) mAb and respective isotype controls were added to the culture when indicated. IFN-γ was measured by ELISA (n = 3-7/group). E, C57BL/6 WT, IL-12p40^-/-^, IL-12p35^-/-^ and IL-23p19^-/-^ neonates were injected with 3.10^5^ CFSE- or VPD450-labeled OT-I T cells, together or not with *Lm* actA^-/-^-OVA. After 60 hours, spleen cells were harvested and OT-I T cell proliferation was analyzed by flow cytometry. Results shown are one representative experiment out of 4. Proliferation Index = MFI_buffer_/MFI_Lm actA_^-/-^-_OVA_ for each mice strain.

We investigated the impact of the IL-12p40 subunit and of IL-10 in the protective immune response against *Lm* infection. Neutralizing anti-IL-12p40 mAb was administered to neonates exposed to *Lm*. The survival rate of neonates was significantly reduced compared to control isotype-treated infected mice (Fig 4A). The impact of IL-12p40 on the primary CD8^+^ T-cell response during *Lm* infection was then assessed. IL-12p40 neutralization during *Lm* actA^-/-^-OVA infection strongly inhibited the frequency of IFN-γ-producing CD8^+^ T cells (Fig 4B) as well as the secretion of IFN-γ (Fig 4C) in response to OVA^257-264^ peptides. Furthermore, when anti-IL-12p40 mAb was added during co-culture of sorted neonatal preCD8α Clec9A^+^DCs with *Lm*-OVA (MOI 1) in the presence of GM-CSF and OT-I T cells, an inhibition of IFN-γ production was obtained (Fig 4D). We further demonstrated that the *in vivo* proliferative response of transferred OT-I T cells in C57BL/6 neonates induced by *Lm* actA^-/-^-OVA infection was significantly inhibited in IL-12p40 ^-/-^ neonates but not in IL-12p35^-/-^ or IL-23p19 ^-/-^ neonates (Fig 4E). Finally, the role played by IL-10 in CD8^+^ T-cell activation induced by *Lm* was assessed by blocking the IL-10 receptor. The IFN-γ production of OT-I T cells in response to *Lm*-OVA presentation *in vitro* by sorted neonatal preCD8α Clec9A^+^ DCs was enhanced in the presence of anti-IL-10R mAb (Fig 4F). These results demonstrated that the IL-12p40 subunit produced by neonatal preCD8α Clec9A^+^ DCs is functional in inducing an efficient primary CD8^+^ T cell response whereas the IL-10 secreted by these precursor DCs moderates the CD8^+^ T cell activation.

### Clec9A antigen targeting to preCD8α Clec9A^+^ DCs in neonates induces efficient protection against *Lm* infection

**Fig 5:**
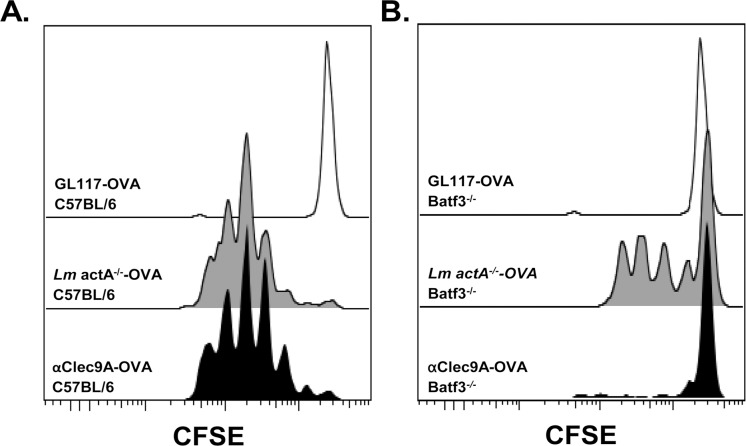
Anti-Clec9A-OVA mAb induces proliferation of OT-I T cells and is specific of CD8α^-^ DCs in neonates. A, C57BL/6 neonates were *i*.*v*. injected with 3.10^5^ CFSE-labeled OT-I T cells, together or not (open black curve) with *Lm* actA^-/-^-OVA (5.10^5^ CFU) (filled grey curve) or anti-Clec9A-OVA mAb (filled black curve). After 60 hours, spleen cells were harvested and OT-I T cell proliferation was analyzed by flow cytometry. Result is one experiment representative of 4. B. Same settings as in (A) in Batf3^-/-^ mice. Result is one experiment representative of 4.

We then investigated whether targeting antigens to Clec9A on the neonatal preCD8α Clec9A^+^ DCs could be an effective strategy for immunization against *Lm* infection. We first tested whether the neonatal preCD8α Clec9A^+^ DCs are able to cross-present to CD8^+^ T cells *in vivo* through Clec9A targeting. C57BL/6 neonates or Batf3^-/-^ neonates were *i*.*v*. injected with either the construct of OVA protein linked to anti-Clec9A antibody (anti-Clec9A-OVA) or with *Lm* actA^-/-^-OVA, along with CFSE-labelled OT-I T cells that were monitored for proliferation. Constructs of OVA linked to an isotype-matched control Ab (GL117-OVA) was used as a control. As seen in Fig 5, anti-Clec9A-OVA administration induced OT-I T cell proliferation *in vivo*, similar to that obtained with *Lm* actA^-/-^-OVA (Fig 5A). Furthermore, the proliferation induced by the Clec9A targeting was exclusively Batf3-dependent, since no proliferation was observed in the Baft3^-/-^ mice compared to GL117-OVA treated mice (Fig 5B) whereas the *Lm* actA^-/-^-OVA-induced OT-I cell proliferation was only partially dependent on Batf3 (Fig 5B). We therefore conclude that neonatal preCD8α Clec9A^+^ DCs serve as effective targets for cross-presentation through Clec9A and for CD8^+^ T cell activation, such as described for MHC-I restricted CD8^+^ T-cell responses in adult mice [30–32].

**Fig 6:**
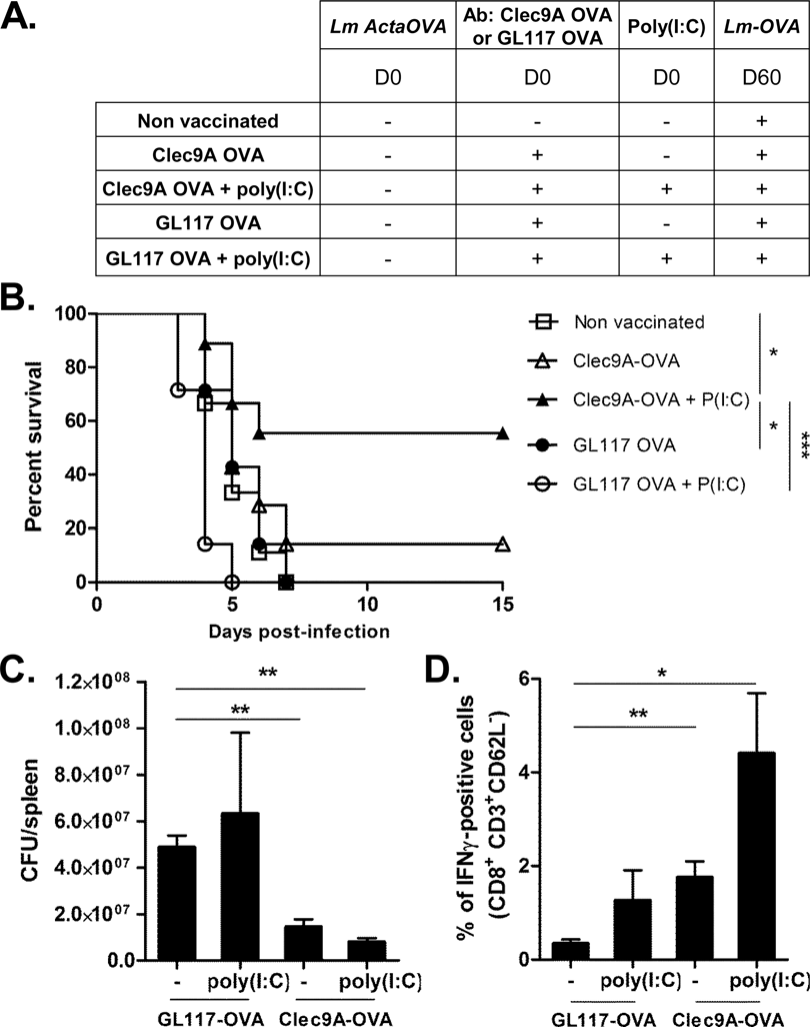
Targeting OVA-antigen to Clec9A on neonatal CD8α^-^ DCs induces protection and efficient CD8^+^ T-cell response to *Lm* infection. A, Immunization protocol for B to D. B, Survival of vaccinated mice, followed during 15 days (n = 12-15/group). C, 3 days after *Lm-*OVA challenge, spleens were harvested and *Listeria* burden was quantified (n = 4/group). D, 5 days post *Lm*-OVA challenge, spleen cells of indicated treated-mice were collected and cultured with IL-2 (10ng/mL) and OVA^257-264^. CD8^+^CD3^+^ cells were analyzed for IFN-γ production by intracellular staining (n = 3-4/group).

In a next step we proceeded to determine whether targeting antigen to preCD8α Clec9A^+^ DCs in neonates by anti-Clec9A-OVA constructs leads to enhanced protection against later *Lm* infection, and whether this requires DC activating agents. Neonates were vaccinated with anti-Clec9A-OVA or GL117-OVA constructs, with or without poly(I:C) as adjuvant. The mice were challenged by infection with *Lm*-OVA 60 days later. A group of unimmunized control mice was infected at day 60 to provide a primary response comparison (Fig 6A). Mice vaccinated at the neonatal stage with anti-Clec9A-OVA and poly(I:C) were partially protected, 55% surviving compared to 90–100% death in control groups (Fig 6B). Immunization with OVA linked to a non-targeting isotype control antibody, or with the targeted construct alone without adjuvant, did not allow survival. The survival of mice that were vaccinated at 3 days of life and challenged 60 days later with L*m*-OVA was correlated with the decrease of bacterial burden. Neonatal immunization with anti-Clec9A-OVA and poly(I:C) reduced the amount of *Lm* in the adult spleen compared to GL117-OVA and GL117-OVA + poly(I:C) (Fig 6C). However, it was surprising to observe a significant early reduction of bacterial load in mice immunized with anti-Clec9A-OVA alone.

We next assessed the efficiency of the secondary T-cell response after vaccination. Five days after infection with *Lm*-OVA, the frequency of splenic IFN-γ producing CD8^+^ T cells in Clec9A-OVA and poly(I:C) immunized group after infection was strikingly higher than in all the other groups (Fig 6D). The use of poly(I:C) during immunization did increase the production of IFN-γ by the T cells after infection. These results with the OVA model antigen system raise the possibility of selectively delivering *Lm* antigens to neonatal preCD8α Clec9A^+^ DCs to trigger a protective immunity against *Lm* infections.

## Discussion

Quantitative and qualitative deficits in the neonatal innate immune response were proposed as causal factors to account for their inability to mount a protective IFN-γ-dependent CD8^+^ T-cell response against various viral pathogens such as respiratory syncytial virus, influenza virus, hepatitis B virus, herpes simplex virus as well as intracellular bacterial pathogens such as *Lm*. Indeed, enhanced neonatal protection against *Lm* infection can be restored through administering recombinant IFN-γ [33], through Flt3L treatment [22] or through administering CpG oligonucleotides [34], both last treatments inducing an IL-12-dependent innate resistance. However, the cellular elements of the neonatal innate immune system able to mount protective type 1 T-cell activation against *Lm* were not clearly identified, as the IL-12p70-producing CD8α ^+^ DC subset was reported to be defective in murine spleen before 7 days of age [2]. It has been well established that CD8α ^+^ DCs play a critical role in mounting an effective cytotoxic CD8^+^ T cell response to *Lm* infection in adult mice [15, 35]. CD8α^+^ DCs are crucial for both efficient bacterial entry into the spleen and induction of the immune response [17, 36].

In this work, we first demonstrated that a murine splenic neonatal CD8α-type DC precursor subset is predominant in the Batf3-dependent DC lineage before 6 days of life. These preCD8α Clec9A^+^ DCs express CD11c^high^, DNGR1/Clec9A, CD24, CD205, and MHCII, without expression of CD8α, B220, CD4 or CD207. They are Batf3-dependent and expand preferentially after a limited Flt3L-treatment (3 days instead of 7 days), compared to pDC or cDC2, which are respectively the transcription factor and the growth factor known to be involved in CD8α^+^ DC subset development [6, 28]. They share the lineage features of CD8α^+^ DCs [37–39] and are most likely converted *in vivo* into CD8α ^+^ DC. These splenic DC precursors are still present in adults but constitute a minor population of the Batf3 lineage as reported by Bedoui et al. who described a similar immediate splenic CD11c^high^,CD24^+^, MHCII^+^, CD205^+^, CD8α^-^ precursor subset capable of cross presenting Ag but poorly addressed their ability to produce cytokines leading to Th1/Tc1 response [40]. We further characterized these adult splenic DC precursors by demonstrating that they were high producers of IL-12p40 exclusively. We also demonstrated that in addition to phagocytosing *Lm*, neonatal preCD8α Clec9A^+^ DCs possess the ability to efficiently cross-present Ag, both *in vitro* and *in vivo*, an ability well described to be restricted to CD8α^+^ DCs [6, 11, 12, 14]. Furthermore, GM-CSF enhances and allows full expression of their capacity to cross-present Ag, similar to the newly formed CD8α lineage DCs in Flt3 ligand stimulated bone-marrow cultures that require a maturation step promoted by GM-CSF to acquire the capacity to cross-present Ag [29]. We therefore conclude that the only CD11c^high^ DCs subset potentially able to cross-present Ag in neonatal spleen are the preCD8α Clec9A^+^ DCs, in contrast to adult spleen in which both the few CD24^+^CD8α^-^ DCs and the predominant CD24^+^CD8α^+^ DCs have this capacity [40].

We clearly demonstrated that neonatal preCD8α Clec9A^+^ DCs are able to produce optimal levels of IL12p40 in response to poly(I:C) and *Lm* without IL-12p70 and IL-23 secretion. This is in accordance with the study which previously described the IL-12p35 gene expression defect of newborn monocyte-derived DC [41]. However, the secretion of IL-12p70 during early life has also been shown to be environment dependent. Indeed, one-day-old purified CD11c^+^ DCs were reported to be able to produce IL-12p70 in response to CpG if a cocktail of maturation agents like GM-CSF and IL-4 were added [23]. Here, we determined that the preCD8α Clec9A^+^ DCs are the subset able to produce IL-12p70 in response to CpG in combination with GM-CSF, IL-4 and IFN-γ. The fact that neonatal preCD8α Clec9A^+^ DCs did not produce IL-12p70 upon *Lm* and poly(I:C) stimulation, even in the presence of GM-CSF, IL-4 and IFN-γ, indicates that they are refractory to maturation through these pathways.

Interestingly, we have shown that IL-12p40 secreted by neonatal preCD8α Clec9A^+^ DCs plays a role in neonatal T cell immunity. The inhibition of IL-12p40 during *Lm* infection increased the neonatal mortality and reduced significantly the Ag-specific CD8^+^ T-cell expansion and activation. Furthermore, the ability of neonatal preCD8α Clec9A^+^ DCs to cross-present OVA Ag *in vitro* or *ex vivo* after *Lm*-actA^-/-^OVA incubation was significantly inhibited when IL-12p40 was neutralized but not IL-12p35 or IL-23p19. Some studies support an independent role for IL-12p40 and more precisely for the IL-12p(40)^2^ homodimeric form. It has been shown that IL-12p40 could act negatively by competitively binding to the IL-12 receptor in an IL-12 mediated shock [42] or by inhibiting IL-23 functions [43]. In contrast, it has also been suggested that IL-12p40 could have a positive role in inducing immune responses [44]. It has been shown that IL-12p40 promotes macrophage inflammation, DC migration and has a protective function in Mycobacterial infection [45, 46]. In line with our findings, it was also demonstrated that IL-12p(40)^2^ was involved in activation of naive T cells and in the induction of IFN-γ production by CD8^+^ T cells [47, 48]. Therefore, IL-12p70 but also IL-12p40 may act as a feed-back loop on costimulatory molecules and MHC molecule expression on dendritic cells to increase naive T cell activation and IFN-γ production [49]. We may therefore conclude that the CD8^+^ T cell immunity induced in early life against *Lm* is IL-12p40 dependent and IL-12p70 or IL-23 independent. These data are in contrast to previous studies showing the requirement of IL-23 in the protection against *Lm* in adults although this role was mostly associated to the activation of IL-17A/IL-17F producing γδ T cells [50] without identifying the cellular source of IL-23. Concerning the role of IL-23 in the CD8^+^ T cell response to *Lm*, it seems to be minor in adult mice [51].

A surprising finding was that, in addition to secreting IL-12p40, neonatal preCD8α Clec9A^+^ DCs produce IL-10. It was already known that neonatal mice display an increased production of IL-10 early in the course of infection with *Lm* and after CpG stimulation, but the source of IL-10 in these neonatal studies had been shown to be macrophages and CD5^+^ B cells [52, 53]. This is the first demonstration that Batf3-dependent DC precursors produce IL-10, suggesting a new mechanism responsible for suboptimal activation of neonatal CD8^+^ T cells. Indeed, blocking IL-10R during cross-presentation after *Lm* stimulation enhanced the production of IFN-γ by Ag-specific CD8^+^ T cells. Numerous publications indicate a regulatory role of IL-10 in DC activation and in the Th1/Th2 polarization in both adults and neonates [52–54]. The direct impact of IL-10 secreted by neonatal preCD8α Clec9A^+^ DCs in T-cell polarization will be analyzed in future investigations.

We demonstrated that these neonatal preCD8α Clec9A^+^ DCs could be used as a cellular target for direct delivery of *Lm* Ags in order to induce efficient immunization when poly(I:C) was co-administrated, allowing a later effective secondary immune response against *Lm* infection. This is in line with previous studies showing that delivering Ags into the cytoplasm of APCs, with for instance synthetic microspheres, was the key for a better induction of neonatal CD8^+^ T cell response [55–57].

Previous studies have shown that DNGR1/Clec9A excels as a target for enhancing CD8^+^ T-cell response and to generating follicular helper T cells in the presence of poly(I:C), in part due to its restricted expression, predominantly in the CD8α^+^ DC lineage and at a lower level in PDC [30, 58, 59]. We observed that injection of anti-Clec9A-OVA construct with poly(I:C) in 3-day-old neonates enhances the frequency of OVA-specific IFN-γ producing CD8^+^ T cells. Furthermore, the absence of CD8^+^ T-cell proliferation in Batf3^-/-^ mice following anti-Clec9A-OVA construct injection confirms a specific involvement of neonatal CD8α^-^ DCs in this process, and argues against a potential role for pDCs. Specifically, we showed here for the first time that a single treatment with anti-Clec9A-OVA construct and poly(I:C) at 3 days of life is enough to significantly enhance the protection of mice against later exposure to the *Lm*-OVA strain. This protective secondary response was associated with a control of the bacterial burden and a memory CD8^+^ T cell response involving Ag-specific IFN-γ producing CD8^+^ T cells. The poly(I:C) treatment was shown to induce *in vitro* the IL-12p40 but not the IL-10 secretion by isolated preCD8α Clec9A^+^ DCs, inhibiting their regulatory properties.

In summary, we have characterized a preCD8α Clec9A^+^ DC subset that is predominant in mouse spleen during the neonatal period. Compared with their adult counterpart or to the adult CD8α^+^ DCs, this neonatal Batf3-dependent DC precursors, that cross-present Ag, display the unique abilities to be high producers of IL-12p40 but also of IL-10. Upon infection with *Lm*, we demonstrated that these preCD8α Clec9A^+^ DCs are endowed with regulatory properties that control the CD8^+^ T cell response through IL-10. The capacity of these neonatal preCD8α Clec9A^+^DCs to induce a protective type 1 T-cell immune response against intracellular pathogens was allowed with anti-Clec9A construct and poly(I:C) treatment through a mechanism involving only the IL-12p40 subunit production with no IL-10.

This discovery opens new strategies for future human vaccine development. It requires the investigation of the ontogeny of the human equivalent of these neonatal preCD8α Clec9A^+^ DCs.

## Methods

### Mice

C57BL/6 CD45.2, C57BL/6 CD45.1, C57BL/6 IL-12p40^-/-^, C57BL/6 IL-12p35^-/-^, Batf3^-/-^ [6] and OT-I TCR transgenic (OT-I) mice were purchased from Jackson Laboratory (Bar Harbor, USA). C57BL/ 6 IL-23p19^-/-^ mice, with EGFP reporter gene, were kindly provided by E. Muraille (Université Libre de Bruxelles, Belgium). Mice were bred and housed in our specific pathogen-free animal facility. For all experiments, neonatal mice are defined as 3-day-old and adults as sex-matched 8-to 12-week-old mice. They were kept in sterile confinement in a P2 animal unit during infections. All animal studies were approved by the institutional Animal Care and Local Use committee.

### Ethics statement

The animal handling and procedures of this study were in accordance with the current European legislation (directive 86/609/EEC) and in agreement with the corresponding Belgian law “Arrêté royal relatif à la protection des animaux d'expérience du 6 avril 2010 publié le 14 mai 2010”. The complete protocol was reviewed and approved by the Animal Welfare Committee of the Institute of Biology and molecular medicine (IBMM) from the Université Libre de Bruxelles (ULB, Belgium) (Permit Number: 2014–43).

### Bacterial strains

*Lm*-EGD strain (*Lm*), Lm-EGD strain deficient for actA ((Lm actA-/-) and *Lm*-GFP strain were kindly provided by Prof. P. Cossart (Pasteur Institute, Paris, France). *Lm*-OVA and *Lm* actA^-/-^-OVA were purchased from DMX incorporated (Philadelphia, PA) [60]. Bacteria were cultured in BBL Brain Heart Infusion (BHI) medium (BD Diagnostics, USA) and stored at -80°C in 10% DMSO.

### Listeriosis model and *in vivo* treatments

For survival studies, mice were injected *i*.*p*. for 7-day-old mice and *i*.*v*. for 3-day-old neonates or adults with different doses of *Lm* diluted in sterile-PBS. To determine the median lethal dose (LD50) of neonates, 3 or 7-day-old and comparative adult C57BL/6 mice were injected with 4 doses (50, 100, 1000 and 10000 CFU) of *Lm* WT and survival rates were observed for 15 days (S8 Fig). All 3-day-old mice died 2 days following 10000 CFU and 5 days following 1000 CFU of *Lm* inoculation; only 20% survived after 100 CFU infections and 60% after 50 CFU. In contrast, adults survived every *Lm* dose administered. The 7 day-old mice showed intermediate sensitivity, dying 8 days after the highest dose of *Lm*, 50% surviving 1000 CFU and all surviving 100 CFU and 50 CFU.

To quantify *Lm* burden, spleens were harvested 3 days after *Lm* infection and homogenized in LPS-free PBS using gentleMACS Dissociator (Miltenyi Biotec, Leiden The Netherlands). Serial dilutions of homogenates were plated on BHI-Agar for 24h at 37°C and bacterial CFUs were assessed.

For primary responses and/or vaccination, neonates mice were *i*.*p*. injected with 5 x 10^5^ CFU of *Lm* actA^-/-^ or *Lm* actA^-/-^-OVA and adult mice were injected with 5 x 10^5^ or 5 x 10^6^ CFU *Lm* actA^-/-^. Neonates were vaccinated *i*.*v*. with 0,1μg of anti-Clec9A/OVA mAb or GL117/OVA control mAb. For secondary responses, 60 days after the first immunization, mice were *i*.*v*. challenged with 5 x 10^5^ CFU of *Lm*-OVA.

When indicated, neonates were injected with neutralizing purified NA/LE Rat anti-Mouse IL-12p40/p70 (clone C17.8; 25μg/neonate 6 hours before, and 1 and 3 days after *Lm* injection) (BD Biosciences) or isotype-matched Ab (BD Biosciences) and with poly(I:C) (1 mg/kg; Sigma-Aldrich). Mice were eventually *s*.*c*. injected with 50μl of saline buffer (NaCl 0,9%) or Flt3L (20μg/ml) at day 0, 1 and 2 of life (Celldex, Phillipsburg, New Jersey).

### Assessment of CD8^+^ T-cell responses

Seven days after primary response or 5 days after *Lm*-OVA challenge, spleen cells were harvested and cultured with OVA^257-264^ peptide (1μg/ml, Polypetides Laboratories, Strasbourg, France) in complete culture RPMI 1640 medium (Lonza Research Products, Switzerland) as described (58). When indicated, IL-2 was added to the culture (10ng/ml, R&D Systems, Minneapolis, USA). Production of IFN-γ by CD8^+^ T-cells was measured by cytometry and ELISA.

### Labeling and *in vivo* proliferation of OT-I T cells

OT-I cells were isolated from lymph nodes of OT-I TCR transgenic mice using the Dynabead untouched mouse CD8 cell protocols (Invitrogen, Life Technologies Europe B.V, Ghent, Belgium). CFSE-labeling (CellTrace CFSE Cell Proliferation Kit, Invitrogen, Life Technologies Europe B.V, Ghent, Belgium) or VPD450-labeling (Violet Proliferation Dye 450; BD Biosciences) were done following the manufacturer’s protocol.

C57BL/6 and Batf3^-/-^ neonates were injected *i*.*v*. with 3x10^5^ unlabeled or CFSE-/VPD450-labeled OT-I cells and with anti-Clec9A/OVA or GL117/OVA construct (0,1μg/mouse) or *Lm* actA^-/-^-OVA (5x10^5^ CFU/mouse). Spleen cells were harvested 60h later. Proliferation of OT-I T cells was assessed on a Cyan ADP cytometer (Dako Cytomation, Everlee, Belgium) by the dilution of CFSE staining.

### Flow cytometric analysis and sorting of DCs

All the following fluorochrome-conjugated mAbs (B220 (RA3-6B2), CD3ε (500A2), CD4 (GK1.5), CD8 (53–6.7), CD11b (M1/70), CD11c (HL3), CD19 (1D3), CD24 (M1/69), CD44 (IM7), CD62L (MEL-14), CD80 (16-10A1), CD86 (GL-1), CD117 (2B8), MHCII (M5/144.15.2) and Sirpα (P84) were purchased from BD Biosciences, fluorochrome-conjugated anti-CD205 mAb (NLDC-45) was purchased from BioLegend and anti-PDCA1 (eBio129c) and CD207 mAb (eBioL31) from ebiosciences (San Diego, CA, USA). Anti-DNGR1 mAb was a kind gift from Dr. Caetano Reis e Sousa (Immunobiology Laboratory, Cancer Research UK’s London Research Institute). For DC characterization, neonatal spleens and lymph nodes from C57BL/6 or Batf3^-/-^ mice were harvested and disrupted using a Pyrex Potter tissue homogenizer (VWR). Red blood cells were lysed by Ammonium-Chloride-Potassium (ACK) Lysing Buffer. Cells (2-5x10^6^) were stained in FACS buffer (PBS/0,5% BSA/2mM EDTA) at 4°C in the dark for 20 min. After fixation in 1% paraformaldehyde (Sigma-Aldrich BVBA, Diegem, Belgium), analysis was performed on a Cyan ADP (Dako Cytomation, Everlee, Belgium).

For DC and CD8^+^ T-cell intracellular staining, splenocytes were incubated at 37°C for 4h in complete culture medium with Golgiplug (1μl/ml; BD Biosciences). Cells were then harvested and stained with extracellular mAbs. Intracellular staining (IFN-γ, clone XMG1.2 and IL-12p40, C15.6) was then done following the manufacturer’s protocol (Cytofix/Cytoperm; BD Biosciences).

For DC sorting and adoptive transfer, spleen cells were first labeled with anti-CD3, -CD19, -B220 and -Gr1 biotinylated mAbs for negative selection using BD IMag Streptavidin Particles Plus DM (BD Biosciences), following the manufacturer’s protocol. Enriched splenocytes were then injected or stained to sort CD11c^high^CD11b^-^CD205^+^CD24^+^ CD8α^-^ DCs (termed CD8α^-^ DCs) or CD8α^+^ DCs (termed CD8α^+^ DCs) on a BD FACSAria II Cell sorter (BD Biosciences).

For phagocytosis assays, spleen cells from neonates were cultured with *Lm*-GFP (MOI 1:5) for 2 hours and stained with CD11c, CD11b, CD205 and CD8α specific mAbs for FACS analysis.

### Cell transfers

30x10^6^ pre-purified C57BL/6 CD45.2^+^ neonatal splenocytes, collected from 20 to 30 neonates by negative selection as described above were *i*.*v*. injected in C57BL/6 CD45.1^+^ adults. Recipient spleen cells were harvested at different time points and were stained to follow CD8α expression on CD45.2^+^ CD11c^+^CD11b^-^CD205^+^CD24^+^ cells by flow cytometry.

### DC function assays

2x10^4^/well of indicated sorted DCs (collected from 40 to 60 neonates and 5 to 7 adults) were cultured for 24h. with CpG (2μg/mL), poly (I:C) (10μg/mL) and *Lm* (MOI 1:1) with or without GM-CSF (20 ng/mL, R&D), IL-4 (20 ng/mL, R&D Systems) and IFN-γ (20 ng/mL, R&D). Supernatants were harvested for cytokine measurements by ELISA.

For antigen presentation assay, sorted CD8α^-^DCs were cultured at 5000 cells/well in complete RPMI-1640 medium at 37°C in the presence of OVA^257-264^-peptide (1μg/mL, Polypeptide Laboratories, Strasbourg, France) or *Lm-*OVA (MOI 1:5) with or without GM-CSF (20ng/ml; R&D Systems), and purified NA/LE Rat Anti-Mouse CD210 (20ng/mL) (IL10R, clone 1B1.3a) or Anti-Mouse IL-12p40/p70 (600ng/mL) (clone C17.8) or isotype matched rat Ig (BD Biosciences). After 4h, isolated OT-I T cells were added to the cultures at a ratio of 1:5 (sorted DC/T). After 48h, IFN-γ was measured by ELISA.

For *ex-vivo* cross-presentation assay, neonates were injected *i*.*p*. with 10^6^ CFU of *Lm-*actA^-/-^ OVA. Neonatal CD8α^-^ DCs were sorted 24h later and co-cultured at 2x10^4^ cells/well with OT-I T cells at a ratio 1:5, with or without GM-CSF (20ng/ml). IFN-γ was measured by ELISA after 48h.

### Quantitation of transcripts and gene expression profiling

For total RNA Quantitative real time PCR and microArrays, neonates and adults were injected or not with 5 x 10^5^ or 5 x 10^6^ CFU of Lm-actA^-/-^ for 24h.

Total RNA from 40,000–60,000 sorted neonatal or adult CD8α^-^ DCs or adult CD8α^+^ DCs (collected from 40 to 60 neonates and 5 to 7 adults) was extracted with phenol/chloroform and purified with the RNeasy microkit (Qiagen) according to manufacturer’s instructions. For quantification of transcripts, reverse transcription and quantitative real-time PCR were performed in a single step using the TaqMan RNA Amplification (Roche Diagnostics) on a Lightcycler 480 apparatus (Roche Diagnostics). For individual samples, mRNA levels were normalized to those of β-actin. Sequence of primers and probes are available on request. For microArrays, total RNA was amplified using the Ovation PicoSL WTA System V2 (NuGen), labeled with biotin using the Encore BiotinIL Module (NuGen), and applied on Illumina HT12 bead arrays at the GIGA-GenoTranscriptomics platform (Liège, Belgium). Microarray data (derived from Affymetrix GeneChip arrays HG-U133 plus 2.0) from CD8α^-^ neonatal and CD8^+^ adult DCs samples were obtained from the National Center for Biotechnology Information Gene Expression Omnibus.

### ELISA

IL-12p40, IL-10 and IFN-γ Duoset ELISA kits and Mouse IL-23 Quantikine ELISA Kit (R&D, Minneapolis, USA) were measured in culture supernatant according to the manufacturer’s instructions. For IL-12p70 ELISA assays, culture supernatant were measured as previously described (23).

### Statistical analysis

Data are expressed as mean ± SEM. Statistical comparison between experimental groups was analyzed using a two-tailed nonparametric Mann-Whitney test for the CFUs, absolute number/% of cells and cytokine levels or with the logrank test for survival curves (GraphPad Prism, GraphPad Software, Inc.). *p* values less than or equal to 0.05 were considered significant. * = p<0,05, ** = p<0,01, *** = p<0,001.

## Supporting Information

S1 Fig Pre-CD8α Clec9A^+^ DCs in lymph nodes of neonates. Cells harvested from lymph nodes of 5-day-old and adult C57BL/6 mice were stained with mAbs and analyzed by flow cytometry for the expression of CD11c, CD11b, CD8α, CD205 and Clec9A. Relative frequencies between CD8α^-^ and CD8α^+^ DCs among CD11c^+^CD11b^-^CD205^+^Clec9A^+^ fraction in neonatal and adult mice are shown (n = 3–4).
(TIF)

S2 Fig Phenotype of neonatal CD8α^-^ and CD8α^+^ DCs and adult CD8α^+^ DCs. A, Neonatal (3-day-old) and adult spleen cells were stained and gated on CD11c^+^CD11b+CD205^+^ CD8α^-^ and CD8α^+^ cells for neonates and on CD8α^+^ cells for adults. Expression of Clec9A, CD24, MHCII, CD86, CD80, CD4, cKIT, CD207 and B220 in these three DCs subsets was analyzed by FACS (grey filled histograms). Open histograms are isotypes staining on respective DCs subsets. Representative of 4 experiments. B, Expression of CD205 and Clec9A on splenic pre-CD8α DCs, pDCs and cDC2 of 3-day-old neonates. pDCs are identified as CD11c^+^CD11b^-^B220^+^PDCA1^+^ cells and cDC2 as CD11c^+^CD11b^+^Sirpα^+^CD4^+^ cells. Representative of 5 experiments.
(TIF)

S3 Fig Analysis of DCs in Batf3^-/-^ and Flt3L-treated mice. A, Dot plot representing the expression of CD11c, CD11b, CD8α, CD205 and Clec9A on spleen cells from C57BL/6 and Batf3^-/-^ 3-day-old neonates out of 4 experiments. B, Frequencies (among total spleen cells) of pDCs and cDC2 in C57BL/6 and Batf3^-/-^ neonates (3-day-old). C, Absolute number of pDCs and cDC2 in control and Flt3L-treated C57BL/6 3-day-old neonates.
(TIF)

S4 Fig Relative expression of genes involved in cross-presentation. Neonatal preCD8α Clec9A^+^ DCs and adult CD8α^+^ DCs were sorted from spleen of neonate (3-day-old) and adult C57BL/6 mice respectively. cDNA was analyzed using Affimetrix GeneChip Arrays. Results are expressed as Log2 Fold Change between CD8α+ (right side) and preCD8α Clec9A+ (left side) DCs (Log2 FC (AD/NN)) for each genes. 3 independent experiments, each coming from 5 adults and 40–60 neonates. Arrows indicate CD8α DCs family gene: CD205, Batf3 and CD24 are equally expressed in neonatal preCD8α DC and adult CD8^+^ DCs except for CD8α as expected.
(TIF)

S5 Fig IL-12p40 production in neonatal and adult CD8α^-/+^ DCs following Poly(I:C) injection. Poly(I:C) was i.v. injected in C57BL/6 neonates and adults (1 mg/kg). Spleen cells were harvested at different time and stained to measure IL-12p40 production in CD8α^-/+^ DCs. Representative of 3–4 experiments for each time point.
(TIF)

S6 Fig IL-23p19 production in neonatal preCD8α Clec9A^+^ DCs following Poly(I:C), *Lm* and CpG stimulation. Sorted neonatal CD8α^-^ DCs were simulated *in vitro* with poly(I:C) (10 μg/mL), *Lm* (MOI 1:1) or CpG (2 μg/ml). IL-4, GM-CSF and IFNγ were added when indicated. IL23p19 was measured by ELISA (n = 4-6/group).
(TIF)

S7 Fig Expression of TLR3 and TLR9 gene in pre-CD8α Clec9A^+^ DCs and CD8α^+^ DCs. mRNA normalized expression of TLR3 and TLR9 gene from preCD8α Clec9A^+^ DCs or CD8α^+^ DCs sorted from spleen of neonates (3-day-old, n = 4) or adults (n = 5) respectively were analyzed by quantitative real-time PCR. Gene expression are presented as normalized crossing point (dCp), obtained by subtracting the Cp of the specific gene from the average Cp of β-actin, used as reference gene.
(TIF)

S8 Fig Age-dependent survival rates after *Lm* infection. Survival of adult, 7-day-old and 3-day-old C57BL/6 mice (n = 10 for adults, n = 10 for 7-day-old and n = 30 for 3-day-old) *i*.*v*. (for adult and 3-day-old mice) or *i*.*p*. (for 7-day-old mice) injected with 10000, 1000, 100 and 50 CFU of *Lm*.
(TIF)
