# Supplementary figures and images for: IL-12p40/IL-10 Producing preCD8α/Clec9A+ Dendritic Cells Are Induced in Neonates upon Listeria monocytogenes Infection
Source: PLoS Pathog. 2016 Apr 13;12(4):e1005561. doi: 10.1371/journal.ppat.1005561 (PMC4830566; doi:10.1371/journal.ppat.1005561)

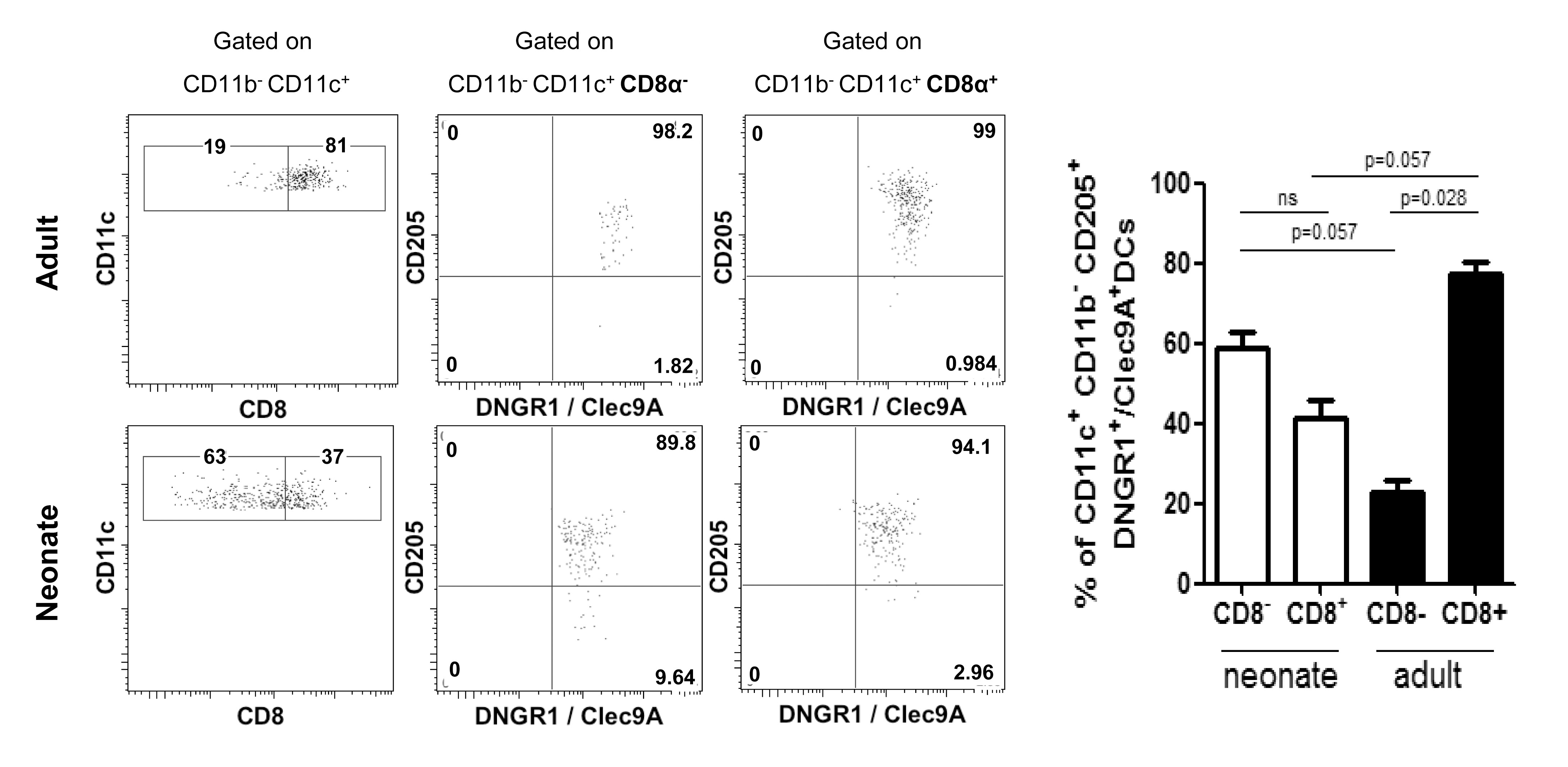

Supplement: S1 Fig — Cells harvested from lymph nodes of 5-day-old and adult C57BL/6 mice were stained with mAbs and analyzed by flow cytometry for the expression of CD11c, CD11b, CD8α, CD205 and Clec9A. Relative frequencies between CD8α- and CD8α+ DCs among CD11c+CD11b-CD205+Clec9A+ fraction in neonatal and adult mice are shown (n = 3–4). (TIF) [file ppat.1005561.s001.tif]

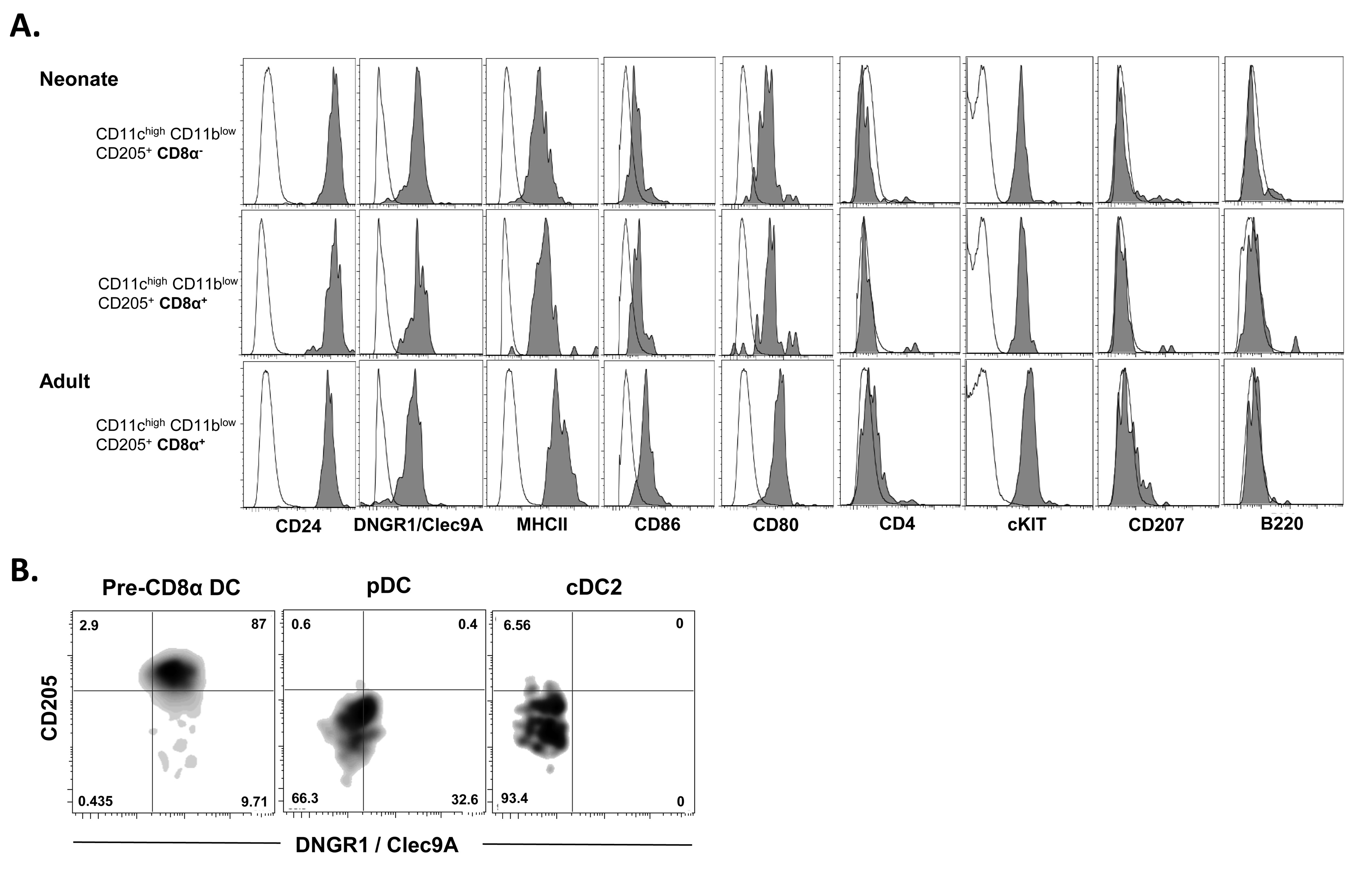

Supplement: S2 Fig — A, Neonatal (3-day-old) and adult spleen cells were stained and gated on CD11c+CD11b+CD205+ CD8α- and CD8α+ cells for neonates and on CD8α+ cells for adults. Expression of Clec9A, CD24, MHCII, CD86, CD80, CD4, cKIT, CD207 and B220 in these three DCs subsets was analyzed by FACS (grey filled histograms). Open histograms are isotypes staining on respective DCs subsets. Representative of 4 experiments. B, Expression of CD205 and Clec9A on splenic pre-CD8α DCs, pDCs and cDC2 of 3-day-old neonates. pDCs are identified as CD11c+CD11b-B220+PDCA1+ cells and cDC2 as CD11c+CD11b+Sirpα+CD4+ cells. Representative of 5 experiments. (TIF) [file ppat.1005561.s002.tif]

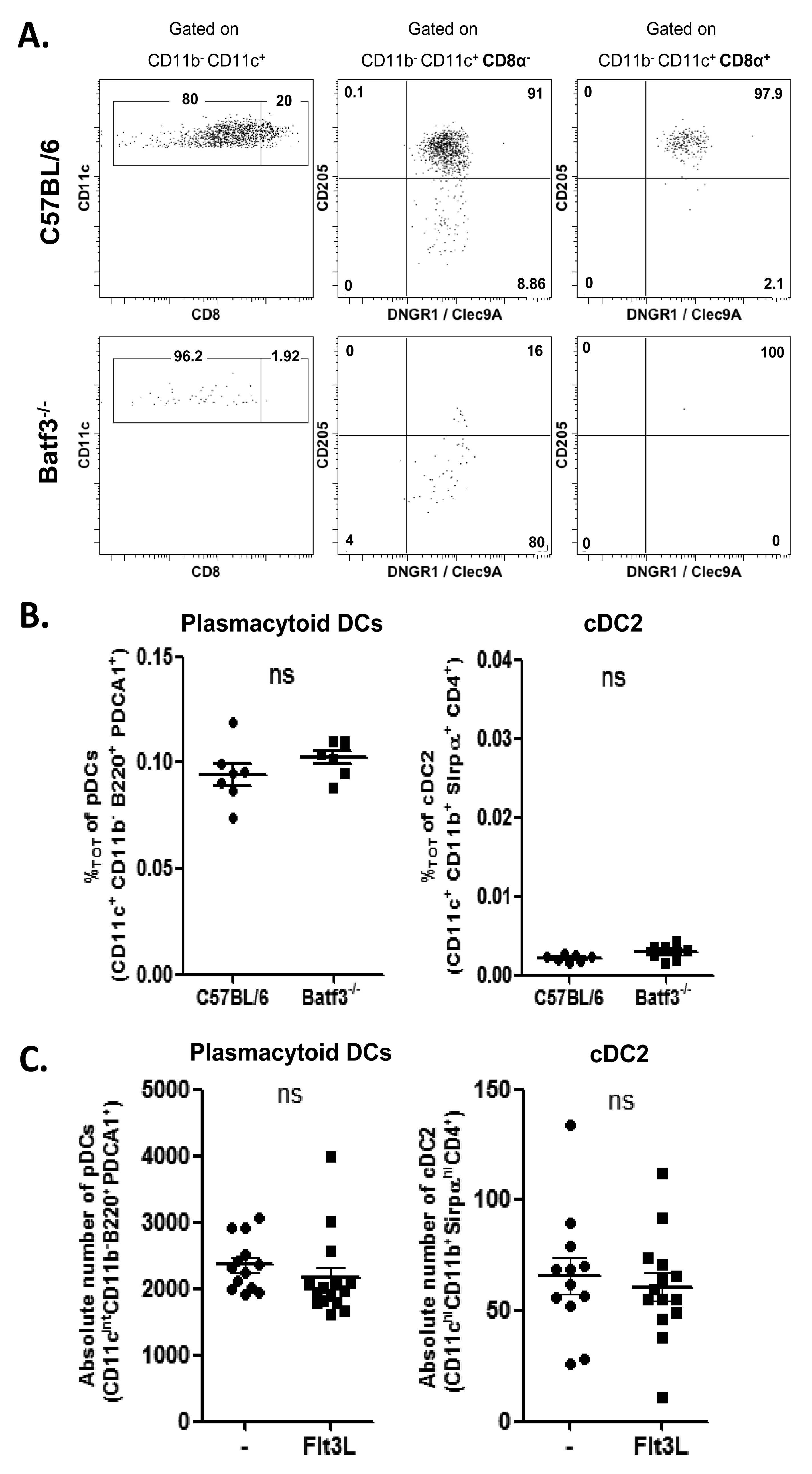

Supplement: S3 Fig — A, Dot plot representing the expression of CD11c, CD11b, CD8α, CD205 and Clec9A on spleen cells from C57BL/6 and Batf3-/- 3-day-old neonates out of 4 experiments. B, Frequencies (among total spleen cells) of pDCs and cDC2 in C57BL/6 and Batf3-/- neonates (3-day-old). C, Absolute number of pDCs and cDC2 in control and Flt3L-treated C57BL/6 3-day-old neonates. (TIF) [file ppat.1005561.s003.tif]

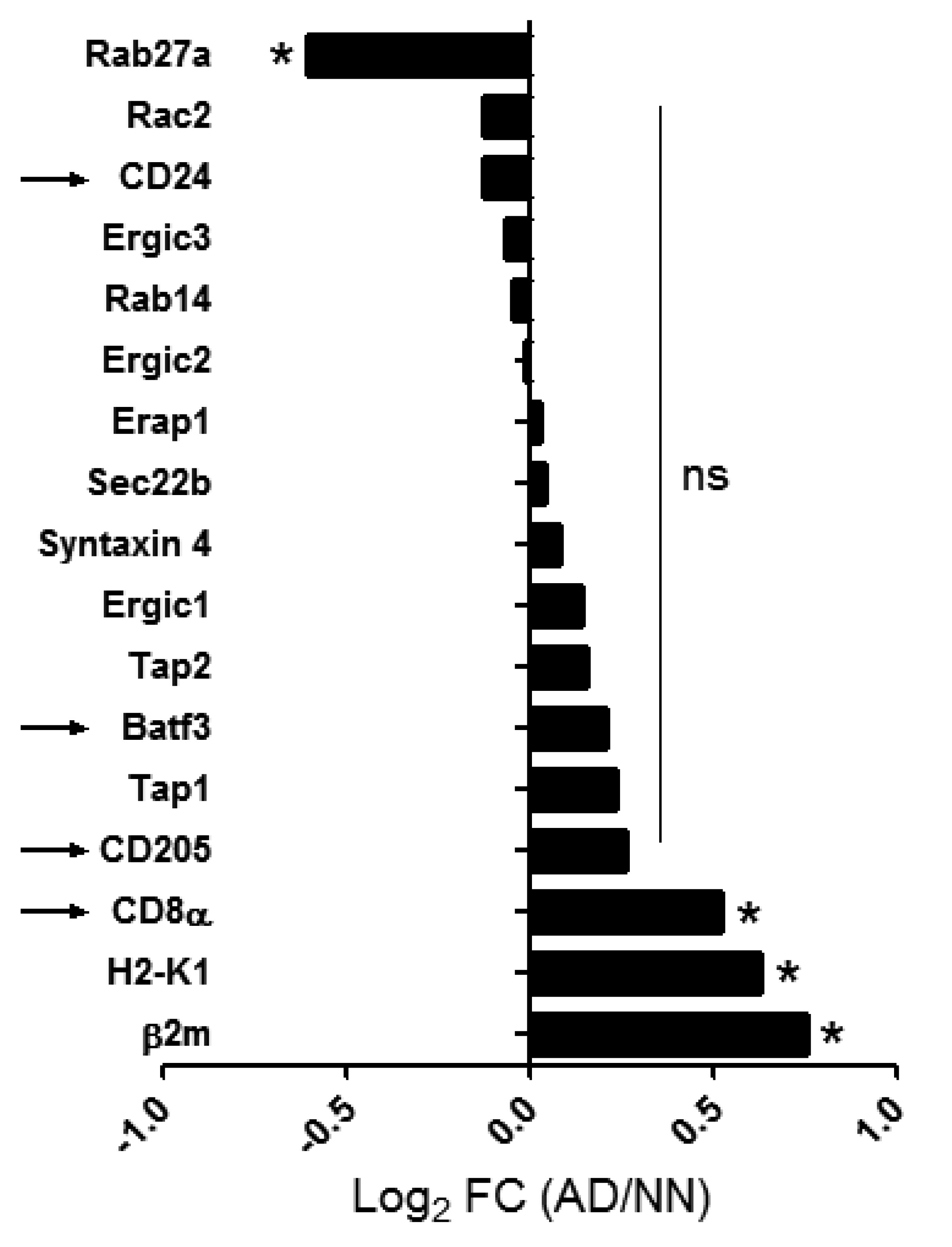

Supplement: S4 Fig — Neonatal preCD8α Clec9A+ DCs and adult CD8α+ DCs were sorted from spleen of neonate (3-day-old) and adult C57BL/6 mice respectively. cDNA was analyzed using Affimetrix GeneChip Arrays. Results are expressed as Log2 Fold Change between CD8α+ (right side) and preCD8α Clec9A+ (left side) DCs (Log2 FC (AD/NN)) for each genes. 3 independent experiments, each coming from 5 adults and 40–60 neonates. Arrows indicate CD8α DCs family gene: CD205, Batf3 and CD24 are equally expressed in neonatal preCD8α DC and adult CD8+ DCs except for CD8α as expected. (TIF) [file ppat.1005561.s004.tif]

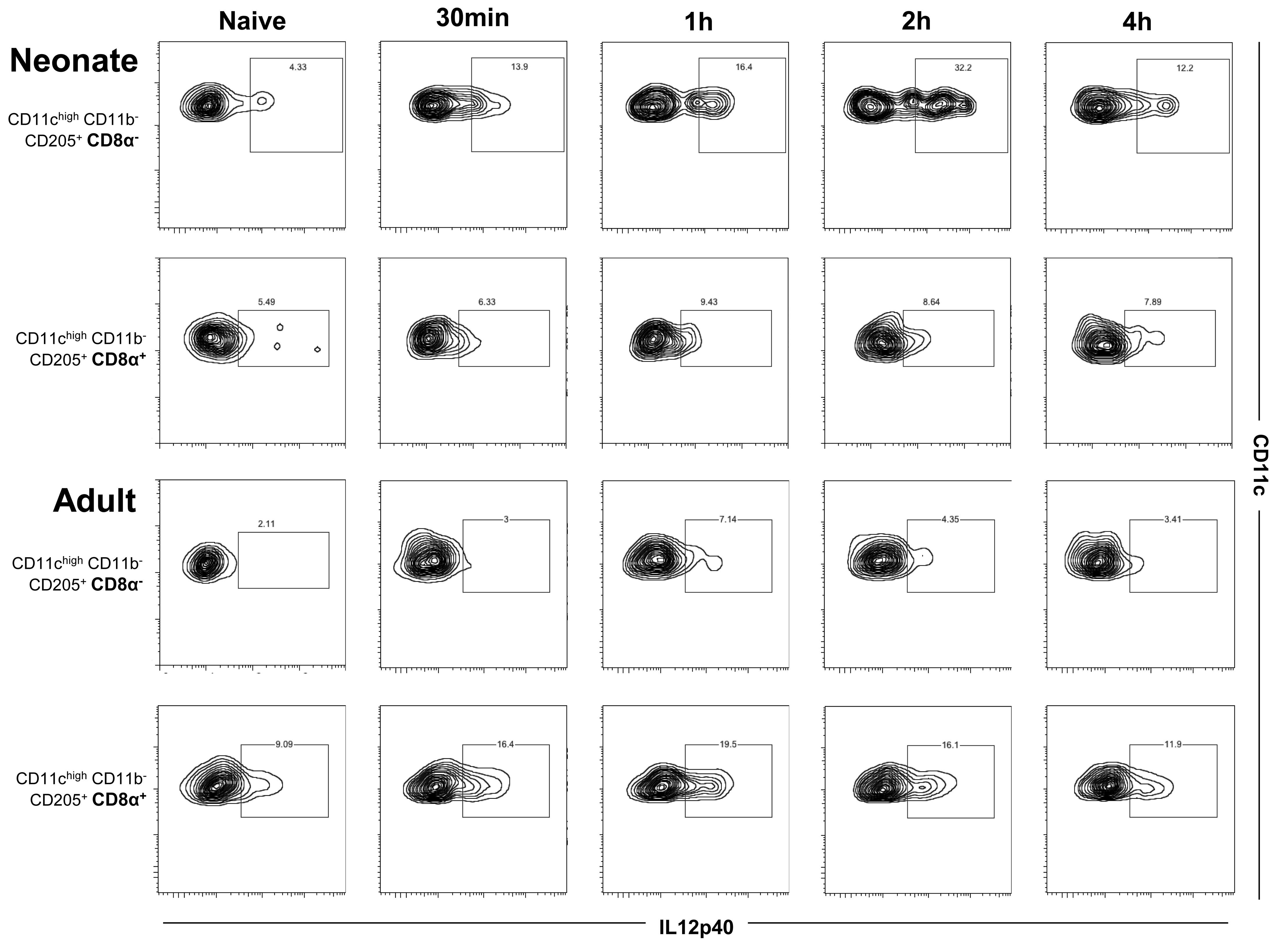

Supplement: S5 Fig — Poly(I:C) was i.v. injected in C57BL/6 neonates and adults (1 mg/kg). Spleen cells were harvested at different time and stained to measure IL-12p40 production in CD8α-/+ DCs. Representative of 3–4 experiments for each time point. (TIF) [file ppat.1005561.s005.tif]

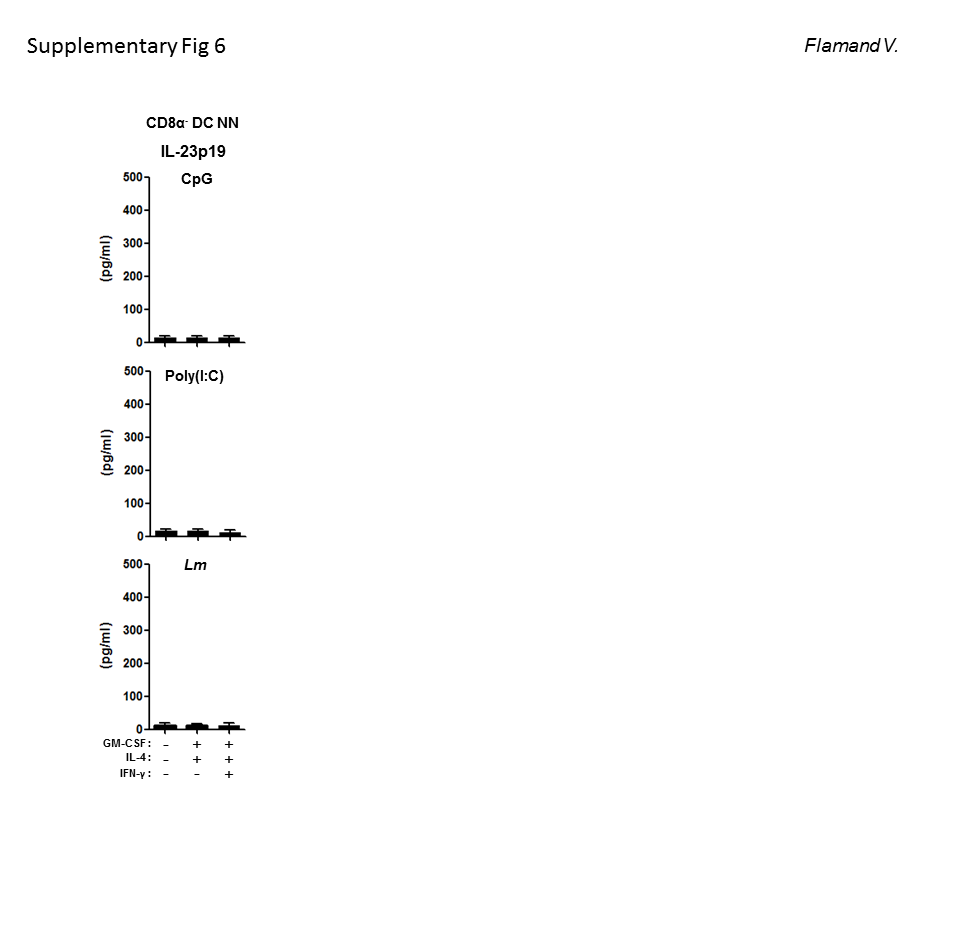

Supplement: S6 Fig — Sorted neonatal CD8α- DCs were simulated in vitro with poly(I:C) (10 μg/mL), Lm (MOI 1:1) or CpG (2 μg/ml). IL-4, GM-CSF and IFNγ were added when indicated. IL23p19 was measured by ELISA (n = 4-6/group). (TIF) [file ppat.1005561.s006.tif]

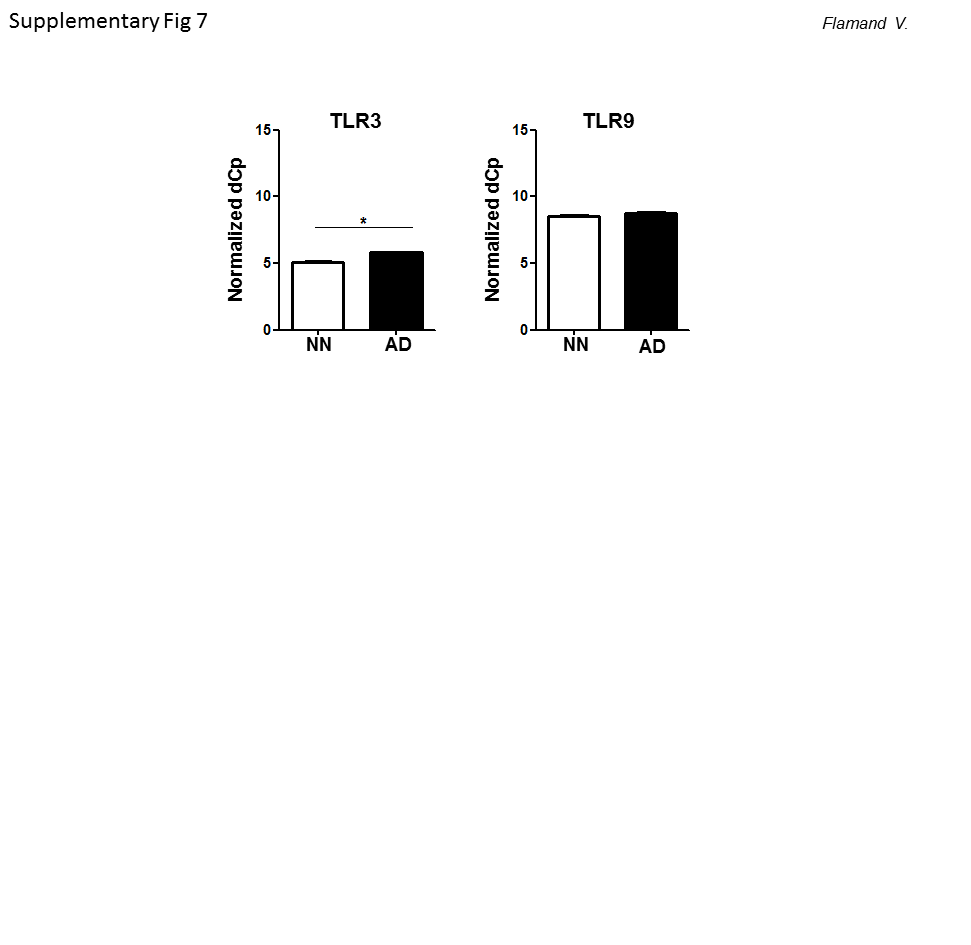

Supplement: S7 Fig — mRNA normalized expression of TLR3 and TLR9 gene from preCD8α Clec9A+ DCs or CD8α+ DCs sorted from spleen of neonates (3-day-old, n = 4) or adults (n = 5) respectively were analyzed by quantitative real-time PCR. Gene expression are presented as normalized crossing point (dCp), obtained by subtracting the Cp of the specific gene from the average Cp of β-actin, used as reference gene. (TIF) [file ppat.1005561.s007.tif]

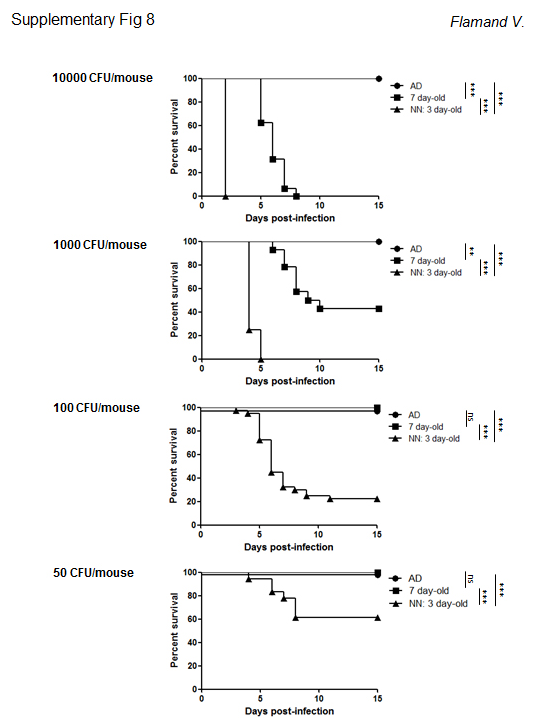

Supplement: S8 Fig — Survival of adult, 7-day-old and 3-day-old C57BL/6 mice (n = 10 for adults, n = 10 for 7-day-old and n = 30 for 3-day-old) i.v. (for adult and 3-day-old mice) or i.p. (for 7-day-old mice) injected with 10000, 1000, 100 and 50 CFU of Lm. (TIF) [file ppat.1005561.s008.tif]
